# FUS-related amyotrophic lateral sclerosis-frontotemporal dementia and links to the DNA damage response: a systematic review

**DOI:** 10.3389/fnmol.2025.1671910

**Published:** 2025-10-31

**Authors:** Seham Almalki, Mohamed Salama, Matthew J. Taylor, Zubair Ahmed, Richard I. Tuxworth

**Affiliations:** ^1^Department of Cancer and Genomic Sciences, School of Medical Sciences, College of Medicine and Health, University of Birmingham, Birmingham, United Kingdom; ^2^Department of Biotechnology, Faculty of Science, Taif University, Taif, Saudi Arabia; ^3^Department of Inflammation and Ageing, School of Infection, Inflammation and Immunology, University of Birmingham, Birmingham, United Kingdom; ^4^Birmingham Centre for Neurogenetics, University of Birmingham, Birmingham, United Kingdom; ^5^University Hospitals Birmingham NHS Foundation Trust, Birmingham, United Kingdom; ^6^Centre for Trauma Sciences Research, University of Birmingham, Birmingham, United Kingdom

**Keywords:** ALS, FTD, ALS-FTD, fused in sarcoma, DNA damage, DDR, DNA repair

## Abstract

Mutations in Fused in Sarcoma (FUS) are associated with neurodegenerative disorders, including amyotrophic lateral sclerosis (ALS) and frontotemporal dementia (FTD). This systematic review examined the connections between DNA damage in the central nervous system (CNS), dysfunction of DNA repair processes and the FUS proteinopathy. Twelve peer-reviewed publications were analyzed, investigating this question across a range of models, including immortalized cell lines, ALS-FTD patient-derived induced pluripotent stem cells, mouse tissues and post-mortem samples from ALS-FTD patients. The studies also explored the impact of inducing DNA damage using several agents, including calicheamicin and etoposide, on FUS pathology. Our findings indicated that accumulated DNA damage was documented in all twelve studies, with a key finding being the disruption of interactions between FUS and the DNA damage response (DDR). FUS interactions with various DDR and DNA repair proteins involved in sensing DNA damage and executing the major repair pathways were impaired, resulting in elevated levels of DNA damage in both the nucleus and mitochondria. Therefore, FUS is an essential protein for the preservation of genomic integrity and this loss of genome stability is likely to be a key contributor to the neurodegeneration in ALS-FTD.

## 1 Introduction

Amyotrophic lateral sclerosis-frontotemporal dementia (ALS-FTD) is a spectrum of neurodegenerative disorders characterized by overlapping clinical and molecular features. ALS primarily affects motor neurons, resulting in progressive degeneration that leads to muscle atrophy and ultimately death due to respiratory failure. In contrast, FTD causes atrophy of the frontal-temporal lobe, leading to impairments in cognitive functions, including behavior and language (Neumann et al., 2009b; Zhou and Xu, 2023). Protein aggregation within the neuronal cytoplasm serves as an indicator of both disorders. Aggregations of Fused in Sarcoma (FUS) and transactive response DNA binding protein 43 (TDP-43) are predominantly associated with ALS, whereas Tau hyperphosphorylation and aggregation, often with accompanying TDP-43 aggregation, is linked to the pathology of FTD (Neumann et al., 2009a; Zhou and Xu, 2023).

FUS is a DNA/RNA binding protein involved in various aspects of DNA and RNA metabolism, including splicing, transport, and the DNA damage response (DDR). Mutations in FUS lead to its aggregation in the cytoplasm and result in mitochondrial dysfunction (Neumann et al., 2009a; Zhou and Xu, 2023). The function of FUS in neurons is of critical importance: neurons are subject to elevated levels of DNA damage due to their high metabolic activity and longevity and in instances of FUS mutations, the proper functioning of the DDR is compromised. This, coupled with elevated reactive oxygen species (ROS) production caused by dysfunctional mitochondria increases the vulnerability of neurons.

Neurons are subject to various forms of DNA damage, including oxidative damage from elevated levels of ROS, as well as DNA strand breaks – both single-strand breaks (SSB) and double-strand breaks (DSB) (Chatterjee and Walker, 2017; Narciso et al., 2016). In post-mitotic neurons, oxidative lesions are predominantly repaired through base excision repair (BER), whereas the error-prone non-homologous end joining (NHEJ) serves as the principal mechanism for the repair of DSB (Chatterjee and Walker, 2017; Narciso et al., 2016). Higher fidelity homologous recombination is generally not available for repair of DSB in post-mitotic neurons where no sister chromatid can be used as a repair template.

While there is currently no FDA-approved treatment for FTD, Riluzole and Edaravone are approved for ALS patients to slow the disease progression by addressing oxidative stress and glutamate toxicity (Tzeplaeff et al., 2023). This underscores the urgent need to identify additional mechanisms that may contribute to the treatment of ALS/FTD.

This systematic review seeks to explore the molecular mechanisms involved in DNA damage and repair within models of FUS-related ALS-FTD, looking across multiple studies and at orthogonal *in vitro* and *in vivo* approaches. Identifying common underpinning factors across disease models will help identify key features that drive disease progression and potentially highlight new therapeutic targets within the DDR and DNA repair processes that can help preserve the integrity of the neuronal genome.

## 2 Methods

### 2.1 Search strategy

**FIGURE 1:**
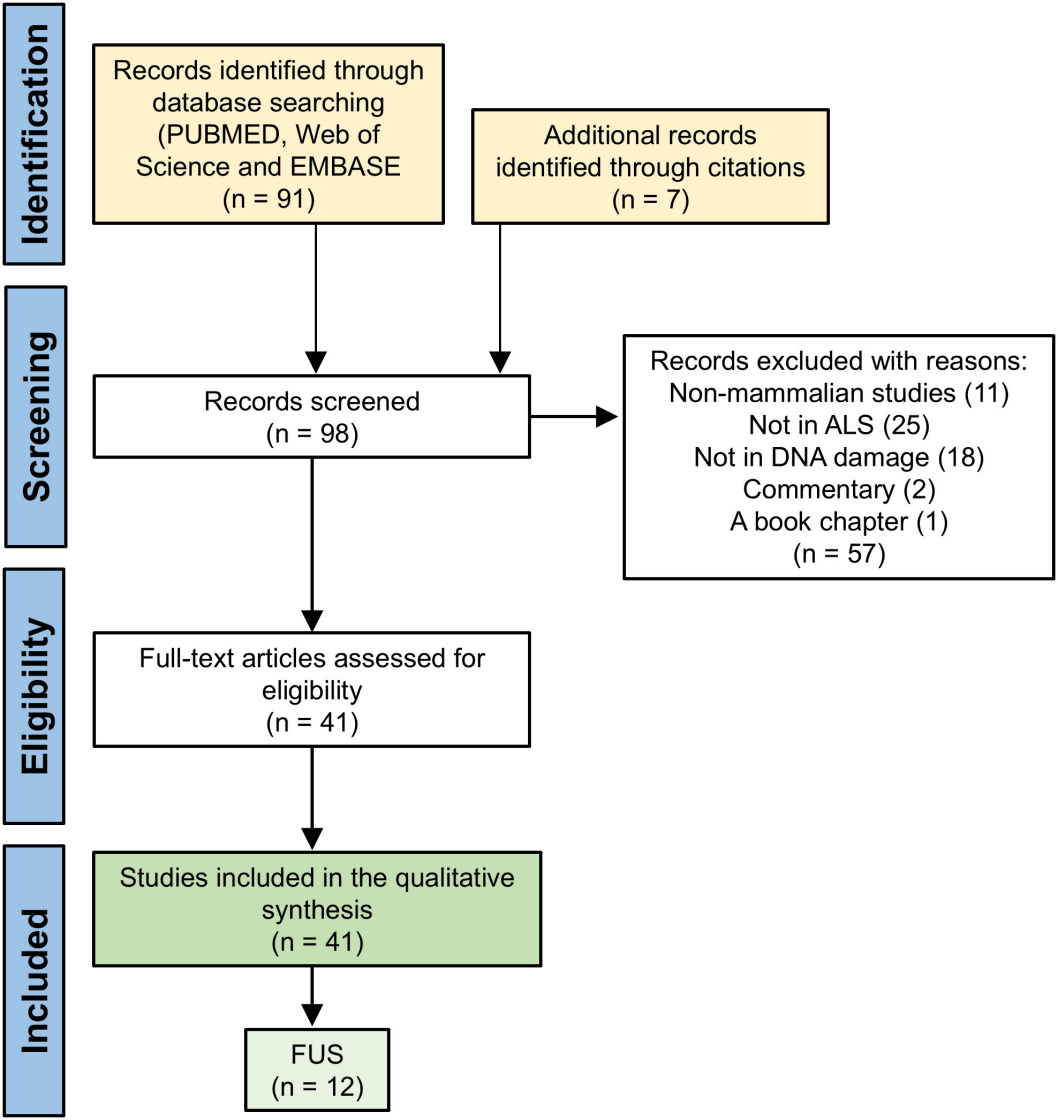
The PRISMA flow diagram indicating the study selection process used in the systematic review.

This systematic review forms part of a wider review which investigates the association between ALS-FTD and DNA damage in the nervous system. The detailed methodology of the review is described in the accompanying article that focusses on ALS-FTD associated with C9orf72 expansions (Almalki et al., 2025). In brief, two authors S.A. and Z.A. conducted a literature search using the Boolean terms “amyotrophic lateral sclerosis” OR “ALS” AND “DNA” AND “double strand breaks” across three databases: PubMed, EMBASE, and Web of Science, in English and published from inception to February 2025. After removing duplicates, we identified 91 remaining studies of which 41 studies were eligible for full-text screening. Twelve articles focused specifically on FUS mutations and proteinopathy in the context of DNA damage, the DDR and DNA repair mechanisms (Figure 1), and hence is the focus of this systematic review. FUS is one component of the FET protein family, and two studies also included data on the other members: EWS1 and TAF15.

## 3 Results

### 3.1 Characteristics of the included studies

The study selection and characteristics are detailed in our accompanying systematic review: C9orf72-related amyotrophic lateral sclerosis-frontotemporal dementia and links to the DDR (Almalki et al., 2025).

### 3.2 Risk of bias (RoB) assessment

**FIGURE 2:**
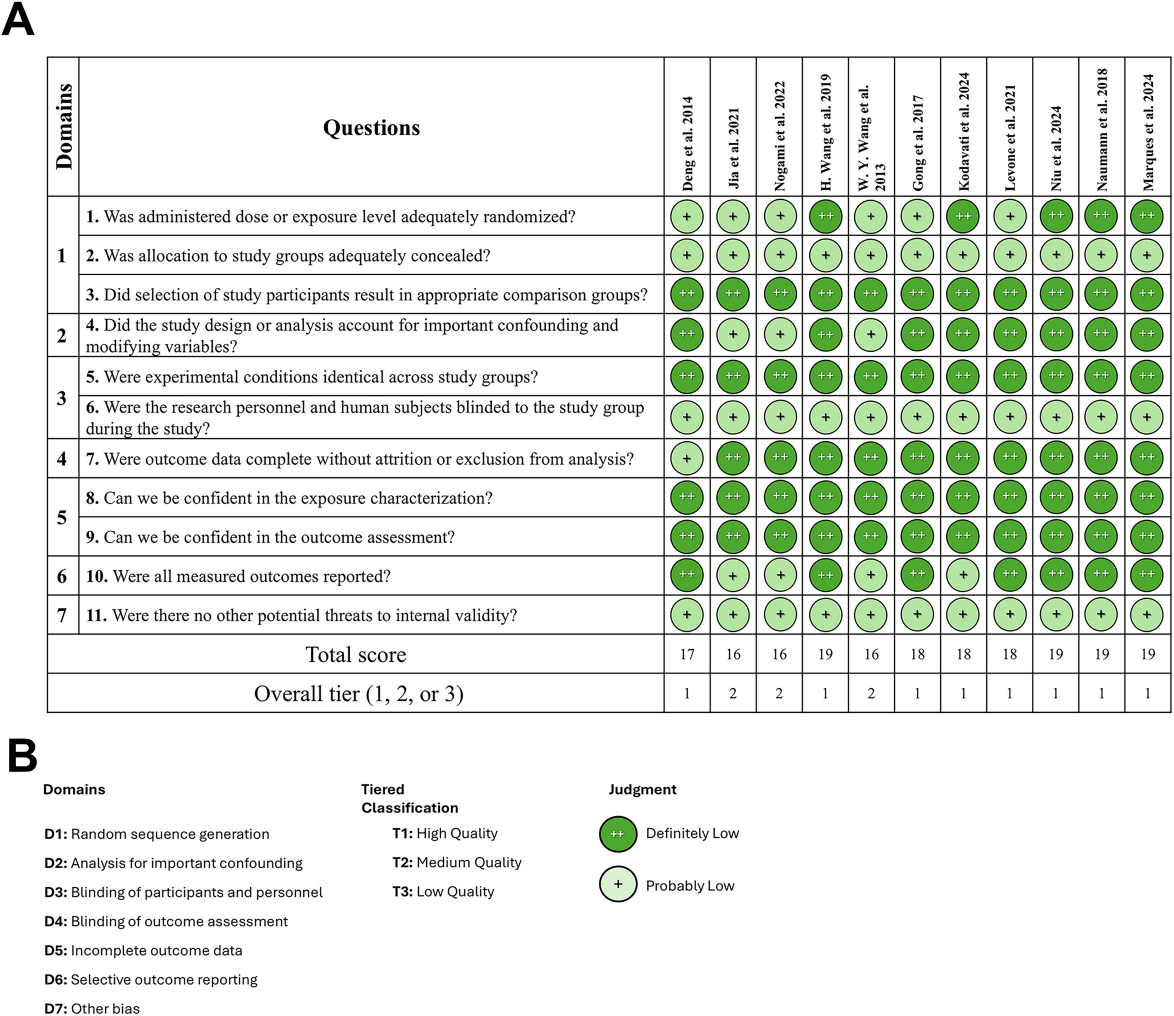
Risk of Bias for *in vitro* studies. The OHAT tool for rating the risk of bias was used for *in vitro* studies. **(A)** Risk of Bias against the questions in the different domains in the included studies. **(B)** Risk of Bias domains and classification criteria.

The Office of Health Assessment and Translation (OHAT) tool (National Institutes of Health, 2019) was used to rate the risk of bias (RoB) of the *in vitro* studies (Figure 2). The tool rates risk of bias across seven domains including randomization, confounding, blinding, outcome data integrity, selective reporting, and other potential biases. Except for one study (de Waard et al., 2010), which did not include *in vitro* experiments, the eleven studies were evaluated using OHAT tool.

Overall, eight out of 11 studies (Deng et al., 2014; Gong et al., 2017; Kodavati et al., 2024; Levone et al., 2021; Marques et al., 2024; Naumann et al., 2018; Niu et al., 2024; Wang et al., 2019) were classified as Tier 1, indicating a low risk of bias and three studies (Jia et al., 2021; Nogami et al., 2022; Wang et al., 2013) were identified as having a moderate RoB and were classified as Tier 2. This suggests generally strong methodological rigor. Domains 5 (incomplete outcome data) consistently showed high confidence across all studies, with all cases rated as “++.”

**FIGURE 3:**
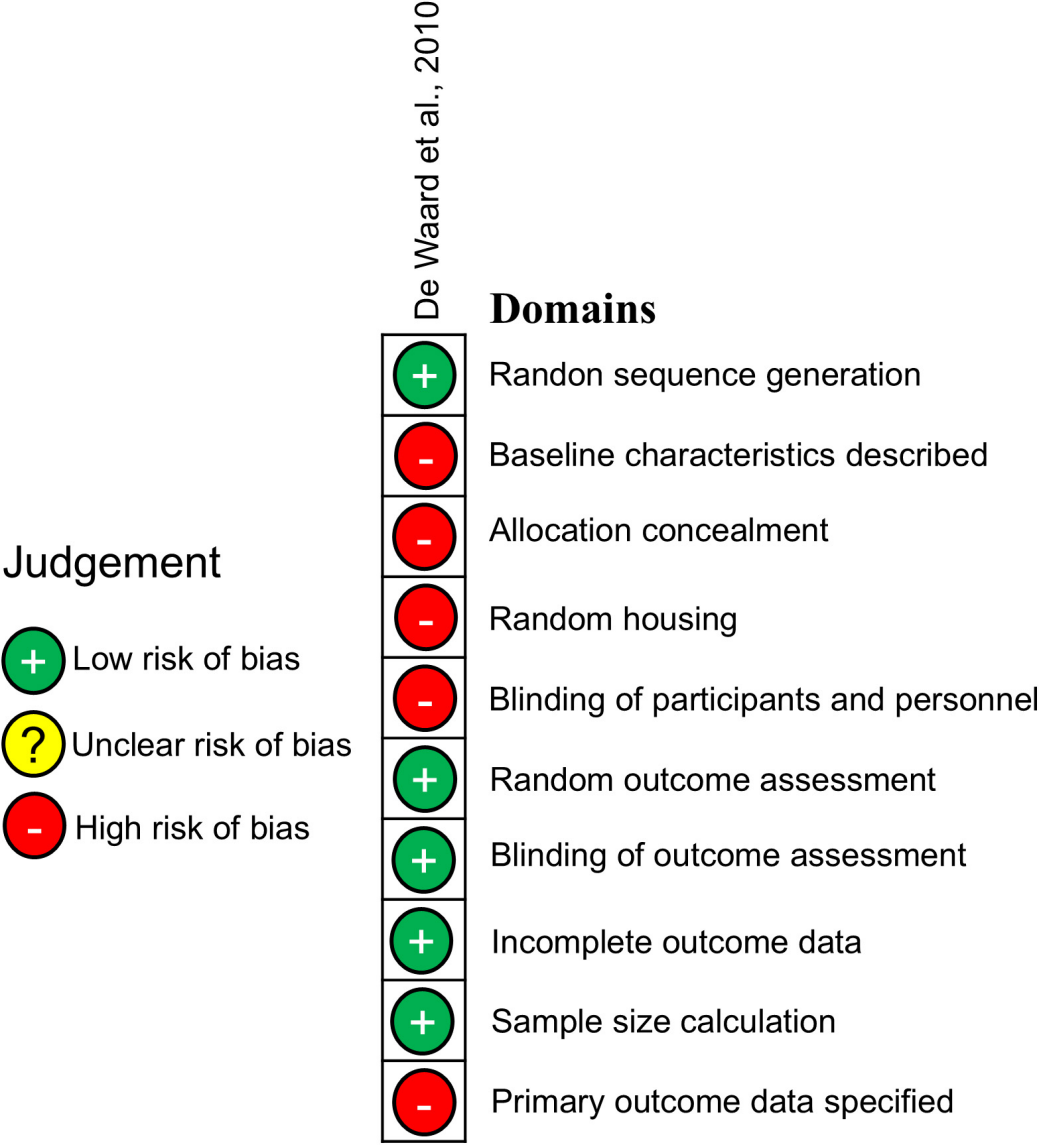
Risk of Bias for *in vivo* studies. The SYRCLE risk of bias assessment was used for the single included *in vivo* study.

The SYRCLE tool (Hooijmans et al., 2014) was used to evaluate RoB in the only *in vivo* study in this review (de Waard et al., 2010; Figure 3). This assessment reveals a high risk in five critical domains, including the baseline characteristics, blinding of participants and random housing. Five domains were assessed as low risk of bias, including incomplete outcome data, and sample size calculation.

In summary, while the *in vitro* studies exhibited an overall low risk of bias, the *in vivo* study showed substantial methodological concerns, particularly in allocation and blinding handling.

### 3.3 Results of studies

The systematic review identified 12 peer-reviewed publications that investigated the role of FUS in DNA damage, DDR, and DNA repair mechanisms. The studies published between 2010 and 2024 exhibit a geographical distribution as follows: six from North America (Deng et al., 2014; Jia et al., 2021; Kodavati et al., 2024; Marques et al., 2024; Wang et al., 2013, 2019), four from Europe (de Waard et al., 2010; Levone et al., 2021; Naumann et al., 2018; Niu et al., 2024), and two from Asia (Gong et al., 2017; Nogami et al., 2022; Table 1). Four experimental models were used, with immortalized cell lines (HEK293, U2OS, HeLa, SH-SY5Y) (Deng et al., 2014; Gong et al., 2017; Jia et al., 2021; Kodavati et al., 2024; Levone et al., 2021; Niu et al., 2024; Nogami et al., 2022; Wang et al., 2013, 2019) being the most prevalent, employed in 9 of 12 cases. This was followed by the use of post-mortem tissues (Deng et al., 2014; Kodavati et al., 2024; Marques et al., 2024; Naumann et al., 2018; Wang et al., 2013, 2019) in 6 of 12 instances, and iPSC-derived neurons from ALS-FTD patients (Deng et al., 2014; Kodavati et al., 2024; Naumann et al., 2018; Niu et al., 2024; Wang et al., 2019) in 5 of 12 cases. Mouse models (de Waard et al., 2010; Kodavati et al., 2024) were the least employed, appearing in only 2 of 12 studies. Nine FUS mutations were investigated: P525L (Kodavati et al., 2024; Naumann et al., 2018; Nogami et al., 2022; Wang et al., 2013, 2019), R244C, R514S, H517Q (Wang et al., 2013), H517D (Nogami et al., 2022), R521C (Gong et al., 2017; Wang et al., 2013), R521H, R495X, and FUS-ΔNLS (Kodavati et al., 2024). Only two studies (Deng et al., 2014; Jia et al., 2021) investigated the role of Ewing Sarcoma (EWS) and TAF15 and, which along with FUS constitute the FET family.

**TABLE 1:** Characteristics of the included studies: FET (FUS, EWSR1, and TAF15) protein family.

References	Country	Model system	Interventions	Research focus	Primary outcomes	Method of detection
**(A) Human and rodent cell line-based studies**						
**Deng et al., 2014**	USA	H4/HEK293T/DDR deficient cells	CLM DDR inhibitors	FTLD-FUS	Cytoplasmic aggregates of FUS due to DNA damage	WB/IP/Kinase assay
**Jia et al., 2021**	USA	U-2 OS	CRISPR-Cas9 DNA damage inducers	FUs function in DNA replication and repair	FUS deficiency causes altered DNA replication timing and DNA damage	IF/WB/RNA-seq/proliferation and cell viability assays
**Nogami et al., 2022**	Japan	U251 MG (H517D or P525L FUS mutations)	CLM DNA-PK inhibitor	DNA damage induces FUS translocation into cytosolic granules	DNA damage stress induces translocation of mutant FUS proteins into cytosolic granules	IF/WB/IP/live cell imaging
**Wang et al., 2019**	USA	HEK293	CRISPR/Cas9/shRNA	FUS role in DDR gene expression	FUS mutations lead to downregulation of key DNA repair genes	RT-PCR array/qRT-PCR/IB
**Wang et al., 2013**	USA	U2OS (FUS-R244C, R514S, H517Q, and R521C)	KD: FUS/BRCA2/LIG4/ DNA damage inducers	FUS role in DDR and DNA repair	FUS mutations impair DNA damage repair through disrupted interaction with HDAC1	IF/IP/ChIP/GFP reporter assay
**Gong et al., 2017**	China	HeLa/U2OS/HEK293T (FUS-R521C)	RNAi/IR (10 Gy)/ DDR inhibitors	RBM45 and FUS interaction in DDR	FUS-R521C enhances interaction with RBM45, leading to impaired DNA damage repair	Co-IP/laser microirradiation imaging/comet assay
**Kodavati et al., 2024**	USA	HEK293	CRISPR/Cas9 Glucose oxidase (GO)	FUS role in mitochondrial DNA repair	FUS mutations impair mtDNA repair, leading to increased mtDNA damage and mutations	IB/LA-PCR/Co-IP
**Levone et al., 2021**	Germany	SH-SY5Y/HeLa	CRISPR CPT/ETO/laser micro- irradiation	FUS role in initiation DDR signal	FUS knockout leads to increased DNA damage and impaired recruitment of DNA repair factors	IF/WB/cell viability assay/Live-cell imaging/HR and NHEJ assays
**Niu et al., 2024**	Germany	Hela	FUS-eGFP UV laser micro-irradiation	FUS recruitment dynamics at DNA damage sites	FUS recruitment dynamics to DNA damage sites differ across cell types	Live imaging
**(B) iPSC-derived motor neurons from ALS patients**						
**Deng et al., 2014**	USA	Cultured astrocytes Cortical neurons	CLM	FTLD-FUS	Cytoplasmic aggregates of FUS due to DNA damage	WB/IP
**Wang et al., 2019**	USA	Fibroblast from fALS FUS-P525L	–	FUS role in DDR gene expression	FUS mutations lead to downregulation of key DNA repair genes	qPCR, IF, IB
**Niu et al., 2024**	Germany	iPSC-derived cells	CRISPR/Cas9 UV laser micro-irradiation	FUS recruitment dynamics at DNA damage sites	FUS recruitment dynamics to DNA damage sites differ across cell types	Cell imaging
**Kodavati et al., 2024**	USA	Spinal Motoneurons and fibroblasts (FUS-R521H and P525L)	CRISPR/Cas9 to correct P525L mutation Glucose oxidase GO	FUS role in mitochondrial DNA repair	FUS mutations impair mtDNA repair, leading to increased mtDNA damage and mutations	LA-PCR/mtDNA sequencing/*in vitro* ligation activity assay
**Naumann et al., 2018**	Germany	Spinal Motoneurons (FUS- P525L)	CRISPR/Cas9 IR/ETO DDR inhibitors	FUS mutations in ALS and DDR	FUS mutations impair DNA damage response signaling, leading to neurodegeneration	WB/ICC/live cell imaging
**(C) Rodent-based studies**						
**Kodavati et al., 2024**	USA	hFUS-R495X transgenic mice FUS overexpression	Deleting NLS	FUS role in mitochondrial DNA repair	FUS mutations impair mtDNA repair, leading to increased mtDNA damage and mutations	WB/LA-PCR
**de Waard et al., 2010**	The Netherlands	Ercc1Δ/- mice SC sections	–	DNA repair deficiency and neurodegeneration	Accumulation of DNA damage plays a key role in neuronal aging and MN vulnerability	IHC/behavioral tests
**(D) Post-mortem brain and spinal cord tissue from ALS patients**						
**Deng et al., 2014**	USA	FTLD-FUS cortical tissues	–	FTLD-FUS	Cytoplasmic aggregates of FUS due to DNA damage	WB
**Wang et al., 2019**	USA	SC tissue	–	FUS role in DDR gene expression	FUS mutations lead to downregulation of key DNA repair genes	qPCR, IB
**Marques et al., 2024**	USA	Cortex and SC tissues from fALS-FUS	–	STING pathway activation in ALS and FTD	DNA damage activates STING pathway in neurons, linked to ALS and FTD pathology	IHC
**Kodavati et al., 2024**	USA	SC tissue	–	FUS role in mitochondrial DNA repair	FUS mutations impair mtDNA repair, leading to increased mtDNA damage and mutations	LA-PCR/mt DNA sequencing
**Wang et al., 2013**	USA	Brain tissues (R521C and P525L mutations)	–	FUS role in DDR and DNA repair	FUS mutations impair DNA damage repair through disrupted interaction with HDAC1	IHC
**Naumann et al., 2018**	Germany	FUS-ALS SC tissue	–	FUS mutations in ALS and DDR	FUS mutations impair DNA damage response signaling, leading to neurodegeneration	IHC

Genes and proteins: FUS, fused in sarcoma protein; HDAC1, histone deacetylase 1; STING, stimulator of interferon genes; Ercc1, excision repair cross-complementing rodent repair deficiency, complementation group 1. Genetic tools and models: KD, knockdown; NLS, nuclear localization signal deletion; hFUS-R495X, human FUS truncated at R495; fALS, FTLD-FUS, frontotemporal lobar degeneration with FUS pathology. Experimental methods: IF, immunofluorescence; WB, western blot; IP, immunoprecipitation; IB, immunoblotting; IHC, immunohistochemistry; ChIP, chromatin immunoprecipitation; Co-IP, co-immunoprecipitation; qRT-PCR, quantitative reverse transcription PCR; RT2 PCR array, targeted gene expression PCR array; RNA-seq, RNA sequencing; LA-PCR, long-amplicon PCR; Live cell imaging, fluorescence-based real-time imaging; GFP reporter assay, green fluorescent protein-based DNA repair reporter assay; Behavioral tests, functional assays for motor and cognitive assessment. DNA damage and repair pathways: DDR, DNA damage response; DSBs, double-strand breaks; mtDNA, mitochondrial DNA; HR, homologous recombination; NHEJ, non-homologous end joining. Chemical reagents and stressors: CLM, calicheamicin (DNA-damaging agent); IR, ionizing radiation; CPT, camptothecin (topoisomerase I inhibitor); ETO, etoposide (topoisomerase II inhibitor); Glucose oxidase (GO), oxidative stress inducer; UV laser micro-irradiation, ultraviolet light–induced DNA damage.

Visual and quantitative methodologies were utilized to investigate FUS recruitment and mis-localization, DNA damage, and DNA repair. The predominant techniques employed include immunofluorescence (de Waard et al., 2010; Deng et al., 2014; Naumann et al., 2018; Niu et al., 2024; Wang et al., 2013), Western blot (Deng et al., 2014; Gong et al., 2017; Jia et al., 2021; Naumann et al., 2018), laser micro-irradiation (Gong et al., 2017; Levone et al., 2021; Niu et al., 2024), and comet assays (Gong et al., 2017; Wang et al., 2013; Table 1).

Two main outcomes were identified from the included studies. The primary outcome was DNA Damage in FUS Models; the secondary outcomes included DDR and DNA Repair Pathway Interactions with FUS, as well as impairment of DNA damage repair.

### 3.4 Primary outcome: DNA damage accumulation

The accumulation of DNA damage was observed in all 12 studies and consistently across the four models (Table 1). Western blot and γH2AX immunostaining assays in a *FUS* knockout HeLa cell line exhibited a significant increase in DSB, with γH2AX levels elevated up to 8.1-fold and (*p* < 0.001), respectively, compared to controls (Levone et al., 2021). Consistent with this, reintroduction of *FUS* into HeLa cells resulted in a 37% reduction in γH2AX foci (*p* < 0.05) when compared to the FUS knockout cells. The accumulation of DNA damage was also observed in brain sections from the motor cortex of familial ALS patients, as indicated by γH2AX staining. DSB were detected in 53% of neurons in post-mortem tissue from patients with a FUS R521C mutation and 61% in sections in patients with a FUS P525L mutation, compared to 20% of neurons in control tissue (Wang et al., 2013). Similarly, a significant increase in γH2AX levels was observed in frontal cortex tissue sample from patients with frontal temporal lobular dementia associated with FUS pathology when compared to controls (*p* = 0.001), along with accumulation of insoluble FUS protein (Deng et al., 2014).

Interestingly, (Naumann et al., 2018) reported that early differentiated iPSC-derived FUS R521C spinal motoneurons exhibited a significant increase in DSB at 14 days *in vitro* (DIV), as indicated by γH2AX staining (*p* < 0.05), when compared to controls, despite no observable FUS mis-localization at this stage. Treatment of mature neurons (30 DIV) with the DNA damaging agent, etoposide, induced a robust increase in γH2AX signal in both control and FUS R521C neurons, with mutant neurons showing significantly higher levels (*p* < 0.05), accompanied by cytoplasmic FUS aggregation.

Several DNA damage agents have been used to induce DNA damage, including calicheamicin (CLM) (Deng et al., 2014; Jia et al., 2021; Nogami et al., 2022), laser-induced damage (Gong et al., 2017; Levone et al., 2021; Niu et al., 2024), and etoposide (Naumann et al., 2018; Wang et al., 2013). We identified distinct patterns in the response of FUS to DNA damage, which exacerbates FUS pathology (Table 3).

### 3.5 FUS and mitochondrial DNA damage

One study Kodavati et al. (2024) reported FUS localization to mitochondria under normal conditions where it plays a key role in maintaining mitochondrial DNA (mtDNA) integrity. Kodavati et al. (2024) demonstrated a significant accumulation of mtDNA damage in multiple FUS models, including following knockout of *FUS* in HEK293 cells (*p* = 0.035) as well as in ALS patient-derived iPSC neurons or fibroblasts and in spinal cord tissue from ALS patients carrying R521H or P525L mutations, (*p* < 0.0001), each compared to controls. Moreover, inducing oxidative DNA damage by expressing glucose oxidase (GO) in the patient-derived neurons or fibroblasts led to increase accumulation and aggregation of FUS P525L protein in mitochondria when compared to the wild-type FUS protein (*p* < 0.0001) (Kodavati et al., 2024).

### 3.6 FUS and the STING pathway

Finally, (Marques et al., 2024) reported activation of the STING pathway, a key mediator of innate immune signaling in response to DNA damage. This occurred selectively in layer V cortical neurons, but not in layer II/III neurons from ALS patients with familial ALS FUS mutations (*p* < 0.01), compared to non-neurological controls.

### 3.7 Secondary outcome: FUS subcellular localization

FUS is primarily a nuclear protein but shuttles between nucleus and cytosol. Three studies (Deng et al., 2014; Naumann et al., 2018; Nogami et al., 2022) report that DNA damage induces changes in FUS localization in a phosphorylation and methylation-dependent process and that some ALS-associated point mutations affect nucleo-cytoplasmic shuttling. Deng et al. (2014) demonstrated an increase in cytosolic FUS protein following induction of DNA damage with CLM in H4 or HEK293 cells that is dependent on DNA-PK phosphorylation of the N-terminus: DNA-PK is a key component of NHEJ DSB repair. Deng et al. (2014) used cell fractionation to quantify nucleus: cytosol ratios of FUS proteins and could detect an increase in cytosolic FUS in human neurons *via* immunofluorescence. Naumann et al. (2018) confirmed that DNA damage generated by treatment with etoposide or arsenite drives some FUS from the nucleus to the cytosol and into foci using iPSC-derived motoneurons expressing FUS-GFP constructs and immunofluorescence. They also demonstrated that the nuclear import of FUS-GFP requires PARP1, which regulates the response to oxidative DNA damage and SSB. A later study by Nogami et al. (2022) failed to show the same effect of CLM treatment on a Venus-tagged FUS protein in U251 cells. Here, an expressed Venus-FUS protein formed large nuclear aggregates rather than translocating to the cytosol. Given nuclear aggregates are not detected in the other studies, this may reflect interference of the Venus tag which was positioned at the critical N-terminus.

Some mutated forms of FUS localize differently than the wild-type form. Many familial ALS FUS mutations are located in the C-terminal domain of FUS which contains the nuclear localization sequence and could potentially affect nucleocytoplasmic shuttling (Wang et al., 2013). In primary mouse, neurons transfected with the familial ALS-associated FUS mutations, R244C or H517Q, exhibited predominantly nuclear expression (Wang et al., 2013). In contrast, neurons expressing FUS R514S or R521C showed both nuclear and cytoplasmic localization (Wang et al., 2013). Naumann et al. (2018) detected foci of FUS P525L-GFP in motoneurons differentiated from iPSCs and then aged, accompanied by increased levels of DNA damage, visualized with γH2AX staining, and degeneration of the distal axons.

Nogami et al. (2022) demonstrated that different mutated forms of FUS can respond differently in response to DNA damage induction. Following treatment with the calicheamicin (CLM), both wild-type and H517D Venus-FUS proteins were predominantly nuclear, but Venus-FUS P525L was distributed in both the nucleus and cytoplasm and was seen to concentrate in cytosolic stress granules. As detailed above, in this study the position of the Venus tag at the N-terminus may have an effect on the nucleocytoplasmic shuttling.

Similarly to FUS, the related proteins of the FET family, TAF15 and EWS, are predominantly nuclear under basal conditions. Treatment with CLM induces translocation of both TAF15 and EWS to the cytoplasm in HEK293 cells and a reduced nuclear-to-cytoplasmic ratio (Table 2). Among the three proteins, the extent of relocation of EWS in response to DNA damage was less than for FUS and TAF15 (Deng et al., 2014; Jia et al., 2021). As for FUS, cytoplasmic translocation of TAF15 and EWS was inhibited by the DNA-PK inhibitor, NU7026, suggesting that the process is dependent on DNA-PK-mediated activation of the DSB repair pathway (Deng et al., 2014).

**TABLE 2:** Characteristics of the included studies: EWS and TAF15.

Study	Country	Model	Interventions	Associated gene	Key findings	Methods
**Human cell line-based studies**						
Deng et al., 2014	USA	HEK293T/Human astrocytes	Calicheamicin 1 (CLM) at 10 nM for 2-3 h	EWS/TAF15	TAF15 mislocation after CLM	Western blot
Jia et al., 2021	USA	H460	Ionizing radiation (2 Gy)	EWS/TAF15	No effect on BRCA1 after IR	Immunofluorescence

### 3.8 Secondary outcome: FUS interaction with the DNA damage response

A complex network links FUS dysfunction to proteins involved in the DDR and DNA repair mechanisms (Table 3 and 4).

**TABLE 3:** FUS-related ALS and associations with DNA damage.

References	DNA Damage Treatment	Consequences for FUS	Cellular response	Statistical significance
Deng et al., 2014	CLM	Accumulation of pFUS in the cytoplasm	Increased γH2AX in FTLD-FUS brains	pFUS: no data found γH2AX: *p* < 0.001
Jia et al., 2021	Hydroxyurea, mitomycin C, camptothecin, calicheamicin1, IR (2 Gy)		Genomic instability and replication stress Altered recruitment and retention of repair factors	↑ genomic instability *p* < 0.05
Nogami et al., 2022	Calicheamicin (10–100 nM, 3.5–5h)	Translocation of FUS-P525L into cytosolic stress granules	Enhanced stress granule formation	↑ FUS mislocalization *p* < 0.001
Wang et al., 2019		Cytoplasmic mislocalization	Downregulation of key DNA repair genes	↓ 9 DDR factors>2-fold
Wang et al., 2013	Eto (5 M, 1 h)	Cytoplasmic mislocalization/phosphorylation	Impaired recruitment of repair factors/↑DNA damage	80%–90% of cells showed nuclear and cytoplasmic FUS localization/ ↓γH2AX (*P* < 0.001)/↓53BP1 *P* < 0.01/DNA damage: ↑*P* < 0.05
Gong et al., 2017	Laser microirradiation	Enhanced interaction with RBM45, decreased interaction with HDAC1 for FUS-R521C	Impaired DNA damage repair	↑ RBM45-FUS interaction: *P* = 0.022–0.029 ↓ HDAC1-FUS *P* = 0.0237 ↓ HR: *P* < 0.0001/JHEJ: *P* = 0.0003
Kodavati et al., 2024	Glucose oxidase (100 ng/ml, 1 h), sodium arsenite	↑ FUS levels in mitochondrial extracts/increased recruitment to mitochondria	↑ mtDNA damage ↓ mtDNA repair	↓ mtDNA integrity*: p* = 0.035 ↓ mtDNA repair *p* > 0.0001
Levone et al., 2021	Camptothecin (0.1 or 0.5 μM, 18 h), ETO (0.5 or 1 μM, 18 h), laser microirradiation	Impaired recruitment to DNA damage sites in FUS-KO cells	↑γH2AX foci, ↓ DNA repair recruitment ↓ Cell viability	↑γH2AX 8.1-fold ↓ KU80 and 53BP1 recruitment ↓ cell viability *P* < 0.001
Niu et al., 2024	UV-A laser micro-irradiation (355 nm)	Altered recruitment dynamics to DNA damage sites	Cell type-specific differences in FUS recruitment and dissociation	greater variability between cell types
Naumann et al., 2018	Eto (2 μM for 72 h)	Cytoplasmic mislocalization/aggregation/recruitment	Increased DNA damage, impaired DDR signaling	↑ FUS mislocalization *p* = 0.001 ↑ DSB *p* = 0.05
de Waard et al., 2010			Accumulation of DNA damage in neurons	No data found
Marques et al., 2024	Eto (5 μM), glutamate (10 μM, 1 h)	-	↑ nuclear γH2AX and cytoplasmic STING levels	↑γH2AX *p* < 0.001 ↑ STING *p* < 0.01

**TABLE 4:** FUS interactions and their effects on DNA repair pathways.

References	FUS Interaction	DNA Repair mechanisms	Results
Jia et al., 2021	53BP1, BRCA1	Homology-directed repair	Reduced recruitment of BRCA1/altered DNA replication timing
Nogami et al., 2022	DNA-PK	–	Abnormal PARP-dependent DNA damage response
Wang et al., 2019	XRCC1, DNA ligase 3	SSR, HR, NHEJ, MMR	BRCA1, LIG4, MSH2, RAD23B downregulation
Wang et al., 2013	HDAC1	HR	Impaired recruitment of repair factors
Gong et al., 2017	RBM45, HDAC1	HR, NHEJ	Impaired HR and NHEJ in FUS-R521C model
Kodavati et al., 2024	mtLig3	mtDNA repair pathway	Increased mtDNA damage/impaired mtDNA repair
Levone et al., 2021	KU80, NBS1, 53BP1	HR, NHEJ	Increased DNA damage/Impaired recruitment of repair factors
Niu et al., 2024	PARP1	HR, NHEJ	FUS recruitment to DNA damage sites is Cell type-vary
Naumann et al., 2018	PARP1, PARG, DNA-PK	Alternative non-homologous end joining (a-NHEJ)	Increased DNA damage/Impaired DDR signaling

### 3.9 Interactions between FUS and the DNA damage response

Four studies (Deng et al., 2014; Naumann et al., 2018; Nogami et al., 2022; Wang et al., 2019) explored the link between FUS and DDR proteins, including three which focused on DNA-PK (Deng et al., 2014; Naumann et al., 2018; Nogami et al., 2022), one on PARP1 (Naumann et al., 2018), and one examining the impact of FUS on the expression of DDR and DNA repair-related genes (Wang et al., 2019). Deng et al. (2014) reported phosphorylation of FUS in response to CLM-induced DNA damage, which was mediated predominantly by DNA-PK activity; phosphorylation was required for shuttling of FUS into the cytosol. Two DNA-PK inhibitors (NU 7026 and NU 7441) diminished the levels of FUS phosphorylation, as measured in Western blots but neither CHK1 and CHK2 inhibitors had any effect, and only partial inhibition was observed with an ATM inhibitor, KU60019. Nogami et al. (2022) identified differential phosphorylation by DNA-PK of wild-type vs. mutated FUS P525L proteins. In human U251 cells treated with CLM, there was a reduction in FUS P525L phosphorylation by about 70% when compared to wild-type FUS.

Naumann et al. (2018) found that PARP1 inhibition in iPSC-derived motoneurons impaired the recruitment of FUS to laser-induced DSB sites (*p* < 0.05), induced cytoplasmic FUS aggregation, and disrupted distal axonal trafficking. While the DNA-PK inhibitor, NU7441, restored FUS nuclear localization (*p* < 0.001), it did not rescue FUS recruitment to sites of DNA damage. FUS also interacts with PARP1, central to multiple types of DNA damage repair. Naumann et al. (2018) showed that PARP inhibitors block recruitment of FUS to laser-induced DSB sites. A mutant FUS P525L protein failed to be recruited to DSB sites but recruitment is restored by PARP inhibition, consistent with a role for PARylation in the localization of FUS to breakpoints.

Finally, focusing on transcriptomics, Wang et al. (2019) reported that depletion of FUS in HEK293 cells led to significant and consistent downregulation (>2-fold) of genes associated with the DDR and with DNA repair, including BRCA1 (3.5-fold), LIG4 (3-fold), MSH2 (4.7-fold), MSH3 (2.9-fold), and RAD23B (4-fold).

### 3.10 FUS interactions with HDAC1

Two studies (Gong et al., 2017; Wang et al., 2013) identified a critical interaction between FUS and HDAC1, a chromatin-modifying enzyme involved in DNA repair. Wang et al. (2013) demonstrated that etoposide-induced DNA damage in primary mouse cortical neurons significantly enhanced the interaction between FUS and HDAC1, with both proteins recruited to DSB sites. In U2OS cells, *FUS* knockdown significantly reduced HDAC1 recruitment to DSB sites when compared to scrambled shRNA controls (*p* < 0.001). Reintroduction of *FUS* combined with knockdown of *HDAC1* expression led to impaired DNA repair efficiency, suggesting these proteins work in partnership (Nogami et al., 2022; Wang et al., 2019). Furthermore, FUS mutations linked to familial ALS (R244C, R514S, H517Q, and R521C) differentially affected the damage-induced interaction with HDAC1. While the R244C, R514S, and R521C mutants failed to show increased interaction following etoposide treatment, the H517Q variant retained a significant damage-responsive interaction (*p* < 0.05) (Wang et al., 2013).

### 3.11 Impairment of DNA damage repair

Impairment in DNA damage repair was reported in various studies (Gong et al., 2017; Levone et al., 2021; Naumann et al., 2018; Wang et al., 2013). Gong et al. (2017) and Levone et al. (2021) demonstrated that FUS mis-localization is associated with impaired recruitment of DNA repair proteins associated with both major DSB repair mechanisms: NHEJ and HR. These include Ku80, 53BP1 and BRCA1, while markers of DNA damage, including of DSB such as γH2AX, were significantly increased, suggesting prolonged damage and reduced efficiency of repair. Wang et al. (2013, 2019) also demonstrated impairment of both DSB mechanisms following knockdown of *FUS* in U2OS cells (HR: *p* < 0.05; NHEJ: *p* < 0.01), which was restored with *FUS* re-expression, and were able to confirm a similar effect on NHEJ in murine primary cortical neurons when *FUS* expression was knocked down (*p* < 0.05) (Wang et al., 2013). As with other phenotypes, different familial ALS-associated FUS mutations had differential effects on NHEJ when expressed in U2OS cells. The R244C, R514S and R521C mutations all caused significant impairment to NHEJ repair efficiency when expressed in U2OS cells and compared to expression of wild-type FUS. In contrast, the R244C and H517Q mutations had no impact on NHEJ (Wang et al., 2013). As described above, (Naumann et al., 2018) identified an increase in DSB in iPSC-derived spinal motoneurons carrying a FUS R521C mutation, when compared to non-mutated neurons, but that γH2AX levels were reduced after a 24-h recovery period (*p* < 0.05), indicating that the cells retain a residual capacity to repair DSB rather than a complete loss of activity.

Mitochondrial DNA (mtDNA) repair is also susceptible to FUS depletion or mutation. (Kodavati et al., 2024) studied mtDNA repair following oxidative stress using an LA-PCR assay. In a *FUS* knockout HEK293 cell line, and in neurons or fibroblasts derived from iPS cells carrying FUS R521H or P525L mutations, mtDNA damage was increased when compared to controls (*p* < 0.0001). Additionally, neural progenitor stem cells differentiated from iPSCs carrying FUS P525L or R521H mutations exhibited significant reduction in FUS-Lig3 interactions, determined by co-immunoprecipitation (co-IP) assays (*p* = 0.002 and *p* = 0.003, respectively), compared to controls.

## 4 Discussion

Our review incorporated 12 peer-reviewed publications to examine the links between DNA and FUS pathology. The results reveal a key function of FUS in preserving the integrity of both nuclear and mitochondrial genomes consistently emerging from multiple methodologies across four biological models. These results point to a relationship in which DNA damage contributes to FUS dysfunction and *vice versa*. The role of FUS in facilitating effective DNA repair through HR and NHEJ was investigated in five distinct studies (Gong et al., 2017; Levone et al., 2021; Niu et al., 2024; Wang et al., 2013, 2019) and two studies examined FUS recruitment to DNA lesion sites and its interaction with HDAC1 (Gong et al., 2017; Wang et al., 2013). Again, largely consistent conclusions across the different studies and models, enables a confident conclusion to be drawn that FUS plays a critical role in maintaining genomic integrity.

Additionally, the presence of DNA damage in the cytosol, resulting from the leakage of nuclear or mtDNA, is sensed by the cGAS-STING pathway. In the current review, only one study investigated STING activation in the context of FUS proteinopathy. Marques et al. (2024) demonstrated that STING activation occurred in cortical neurons from ALS patients carrying FUS mutations. Similarly, activation of the STING pathway has been increasingly identified as a common mechanism in several neurodegenerative disorders beyond ALS-FTD. For example, Hinkle et al. (2022) showed that in an α-synucleinopathy mouse model of Parkinson’s disease, STING activation promoted neurodegeneration. (Gulen et al., 2023) reported that neurodegeneration and cognitive decline in aged mouse brains were associated with microglial STING signaling, and that blocking of cGAS–STING signaling attenuated these effects. In Alzheimer’s disease, (Quan et al., 2025) reviewed evidence that cGAS-STING pathway was activated in response to leakage of mtDNA in experimental models. Collectively, these findings suggest that the STING pathway is a common downstream mediator of DNA damage in multiple neurodegenerative disorders. Importantly, while (Marques et al., 2024) provided evidence for STING activation in cortical neurons of ALS patients, studies in Parkinson’s disease (Hinkle et al., 2022) and Alzheimer’s disease (Quan et al., 2025) primarily focused in microglia cells to show the activation of STING pathway. This indicates that STING contributes to neurodegeneration through both neuronal and glial mechanisms. Since neurons are more susceptible to DNA damage, further research is necessary to specifically investigate STING activity in neurons during neurodegeneration.

Our review revealed a consistent picture that not all ALS-associated FUS mutations act in the same manner. Mutation-specific differences in response to phosphorylation of FUS by DNA-PK and which can lead to its cytoplasmic mis-localization were uncovered (Deng et al., 2014; Nogami et al., 2022). The differential phosphorylation effect suggests that specific FUS mutations may impair DNA-PK signaling pathways to varying degrees, potentially altering cellular susceptibility to DNA damage between patients. The molecular profiling of individuals may be able inform therapeutic decision-making in ALS-FTD in the future.

Additionally, in this review we highlighted the role of DNA-PK in phosphorylating FUS in response to DNA DSBs. Importantly, phosphorylation of FUS by DNA-PK can also occur in the absence of DNA damage as a post-translational modification, indicating a non-canonical function of DNA-PK beyond its classical role in DSB repair. This suggests a strong link between the DDR and the regulation of FUS aggregation, thereby connecting genome instability with FUS proteinopathy. An *in vitro* study by Monahan et al. (2017), not included in our systematic review, demonstrated that DNA-PK phosphorylates the low complexity domain of FUS, which reduces the formation of aberrant cytosolic aggregates characteristic of ALS-FTD pathology. The study showed that phosphorylation or phospho-mimetic substitution of the multiple S/TQ motifs within the low complexity domain, introduces negative charges that disrupt liquid-liquid phase separation, a process underlying aberrant FUS aggregation. This finding is important because it suggests that targeting DNA-PK could have therapeutic potential not only to preserve genomic integrity but also to prevent FUS aggregation and mislocalization.

One of the key findings highlighted in this review is the early detection of DNA DSB reported by (Naumann et al., 2018). They observed a significant increase in γH2AX levels in iPSC-derived FUS R521C spinal motoneurons as early as 14 DIV, even in the absence of cytoplasmic FUS mislocalization. If these findings translate from *in vitro* to patients, it suggests that DNA damage accumulation is likely to be an early event in the disease course, prior to the onset of symptoms, and potentially may trigger a feedforward cycle of FUS dysfunction and further DNA damage. Further studies are needed to investigate early DNA damage events across ALS-FTD subtypes and models.

## 5 Conclusion

Fused in Sarcoma plays a crucial role in maintaining genomic integrity. Mutations or downregulation of FUS result in altered protein localization, an increase in DNA damage, impaired DNA repair mechanisms, and compromised interactions with key DNA repair factors such as HDAC1 (Gong et al., 2017; Wang et al., 2013) and PARP1(Naumann et al., 2018; Niu et al., 2024; Nogami et al., 2022). Furthermore, FUS is involved in regulating mtDNA repair and the activation of the STING pathway (Marques et al., 2024). This combination of events underpins neurodegeneration in cases of ALS-FTD.

## 6 Limitations

While this systematic review collates substantial evidence highlighting the importance of DNA damage and impaired DNA damage repair in models of FUS-related ALS-FTD, several limitations need to be considered. First, although employing a variety of DNA damage treatments allows responses to different insults to be investigated, comparing results across models proved challenging due to the heterogeneity among these treatments, which included chemical agents and laser microirradiation, as well as variations in the timing and dosages used. Second, the absence of behavioral data in animal models highlights an urgent need to investigate FUS pathology and DNA damage in animal behavior and explore the long-term consequences. All of the included studies focused on *in vitro* models with the exception of one: (de Waard et al., 2010). The OHAT tool showed low risk of bias in these *in vitro* studies which, taken together with the consistency of findings between studies and across models, provides confidence in our conclusions. Conversely, RoB analysis of (de Waard et al., 2010) using SYRCLE reveals a high RoB in key domains – primarily blinding of participants and random housing. However, the primary focus of this paper is not the investigation of FUS pathology, although it does include related findings. Consequently, the high RoB for this paper has little impact on the overall conclusions presented here.
